# Mitochondria and cell death-associated inflammation

**DOI:** 10.1038/s41418-022-01094-w

**Published:** 2022-11-29

**Authors:** Esmee Vringer, Stephen W. G. Tait

**Affiliations:** 1 Cancer Research UK Beatson Institute, Glasgow, UK; 2 Institute of Cancer Sciences, University of Glasgow, Glasgow, UK

**Keywords:** Cell biology, Autophagy, Cancer metabolism

## Abstract

Mitochondria have recently emerged as key drivers of inflammation associated with cell death. Many of the pro-inflammatory pathways activated during cell death occur upon mitochondrial outer membrane permeabilization (MOMP), the pivotal commitment point to cell death during mitochondrial apoptosis. Permeabilised mitochondria trigger inflammation, in part, through the release of mitochondrial-derived damage-associated molecular patterns (DAMPs). Caspases, while dispensable for cell death during mitochondrial apoptosis, inhibit activation of pro-inflammatory pathways after MOMP. Some of these mitochondrial-activated inflammatory pathways can be traced back to the bacterial ancestry of mitochondria. For instance, mtDNA and bacterial DNA are highly similar thereby activating similar cell autonomous immune signalling pathways. The bacterial origin of mitochondria suggests that inflammatory pathways found in cytosol-invading bacteria may be relevant to mitochondrial-driven inflammation after MOMP. In this review, we discuss how mitochondria can initiate inflammation during cell death highlighting parallels with bacterial activation of inflammation. Moreover, we discuss the roles of mitochondrial inflammation during cell death and how these processes may potentially be harnessed therapeutically, for instance to improve cancer treatment.

## Facts

- MOMP is inherently pro-inflammatory.
- Apoptotic caspase activity inhibits cell death-associated inflammation.
- Mitochondrial-derived DAMPs can be bacterial-like.
- Mitochondrial-driven inflammation can enhance the immunogenicity of cell death.

## Open questions

- Is mitochondrial-driven inflammation during cell death driven by bacterial-like DAMPs?
- How do mitochondria release immunostimulatory mtDNA?
- Can inflammation occur under caspase-proficient conditions?
- What physiological functions has mitochondrial inflammation during cell death?

## Introduction

Mitochondria, with rare exceptions, are found in all eukaryotic cells. Amongst their many roles, mitochondria play a crucial function in energy production, iron homeostasis, and the biosynthesis of lipids, amino acids and nucleic acids [1, 2]. In addition, mitochondria harbour many damage-associated molecular patterns (DAMPs) that can initiate a variety of inflammatory signalling pathways [3]. Some of these mitochondrial DAMPs share similarities with pathogen-associated molecular patterns (PAMPs) found in bacteria and may be derived from their bacterial ancestors. Approximately 1.5 billion years ago endosymbiosis between archaebacteria and a prokaryotic cell, driven by increased oxygen levels, led to the formation of mitochondria that we know nowadays [4]. As a result of this, mitochondria incorporated transport proteins, acquired cristae structure, and integrated metabolic pathways and fission-fusion machinery with the host cell thereby providing an evolutionary advantage compared to prokaryotic cells [5, 6]. Many parallels can be drawn between mitochondria and bacteria including their morphology. Mitochondria and bacteria also harbour circular DNA containing CpG-rich motifs. Furthermore, gram-negative bacteria and mitochondria both have a double phospholipid membrane layer—the inner and outer membrane. The inner membrane encapsulates the cytosol of gram-negative bacteria and the matrix of mitochondria and is rich in the phospholipid cardiolipin. The space between the two membranes is referred to as the periplasmic space in gram-negative bacteria and the intermembrane space in mitochondria [6].

**Fig. 1:**
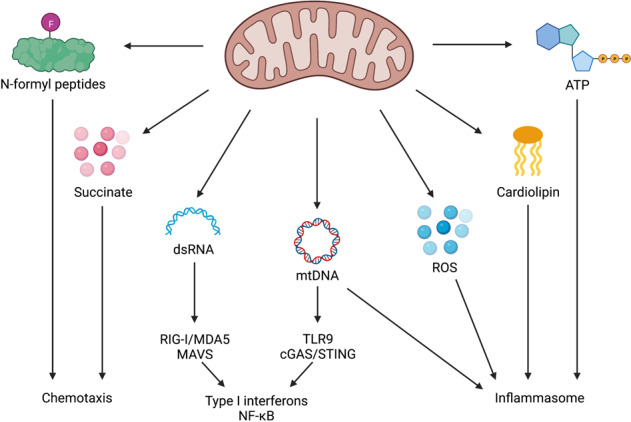
Overview of mitochondrial-derived DAMPs. Mitochondria contain DAMPs that can be exposed upon mitochondrial stress and damage. These DAMPs include succinate, N-formyl peptides, dsRNA, mtDNA, ROS, cardiolipin, and ATP. Loss or exposure of these DAMPs activates several immune pathways including transcription of type I interferons and NF-κB target genes, inflammasome activation, and the recruitment of immune cells.

Mitochondria contain several DAMPs that can be released upon mitochondrial stress or damage (Fig. 1). These DAMPs include mitochondrial DNA (mtDNA), cardiolipin, N-formyl peptides (NFPs), and reactive oxygen species (ROS) but also metabolites such as adenosine triphosphate (ATP) and succinate [3]. NFPs are mainly found in bacteria where formyl modified methionine initiates protein synthesis [7]. These NFPs are a chemoattractant for host phagocytes and can be recognised by formyl peptide receptors on the plasma membrane [8, 9]. Due to its bacterial ancestry, mitochondrial formylation of methionine is required for translation initiation of mtDNA-derived mRNA [7] and will therefore be recognised by the same formyl peptide receptors. In addition, detection of the extracellular mitochondrial metabolite succinate has shown to enhance the immune response of dendritic cells [10]. mtDNA is one of the most investigated DAMPs over the past few years, especially in the context of mitochondrial apoptosis. The striking similarities between mtDNA and bacterial DNA leads to its recognition by endosomal Toll-like receptor 9 (TLR9) [11, 12]. In addition, cytosolic DNA from any source can be detected by the 2’3’-cyclic GMP-AMP (cGAMP) synthase (cGAS) leading to an interferon type I response by the stimulator of interferon genes (STING) [13–15]. A third mitochondrial DAMP shared with bacteria is the phospholipid cardiolipin. Exposure of mitochondrial cardiolipin can lead to mitophagy where it functions as an ‘eat-me’ signal [16]. Several studies imply a role for cardiolipin in mitochondrial apoptosis by facilitating BAX pore formation especially in the context of tBID [17–19], however the extent by which cardiolipin is required for mitochondrial apoptosis is highly variable. In addition, cardiolipin is also known to induce NLRP3 inflammasome activation upon the presence of PAMPs or antibiotics [20, 21]. Furthermore, the NLRP3 inflammasome can also be activated by mtDNA and mitochondrial-derived ROS [22–25].

In this review, we discuss mitochondrial-driven inflammation during cell death and how this is silenced during apoptosis. Furthermore, we speculate about potential new mitochondrial-driven immunogenic pathways based on bacterial similarity. Finally, we discuss how mitochondrial-dependent inflammation may be therapeutically exploited to improve cancer treatment.

## Apoptosis—an immunosilent form of cell death

**Fig. 2:**
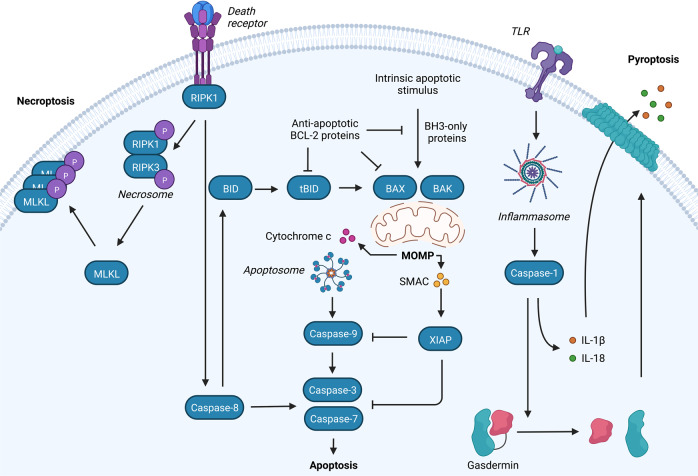
Overview of necroptotic, apoptotic and pyroptotic signalling pathways. Binding of ligands to one of the death receptors (e.g. TNF or Fas) initiates pleiotropic signalling leading to cell survival, inflammation, apoptosis or necroptosis, mediated by the key signalling protein RIPK1. Upon caspase-8 inhibition, RIPK1 forms the necrosome with RIPK3 leading to phosphorylation and activation of MLKL causing membrane permeabilization and necroptosis. Activation of initiator caspases (caspase-8) by death receptors leads to cleavage and activation of executioner caspases (caspase-3 and -7) causing apoptosis. The intrinsic pathway requires an intrinsic apoptotic stimulus which activates pro-apoptotic BCL-2 family members BAX and BAK. Upon their activation pores are formed in the mitochondrial outer membrane leading to the release of intermembrane space proteins (including cytochrome *c* and SMAC). Release of cytochrome *c* allows apoptosome formation which recruits and activates caspase-9 followed by the activation of the executioner caspases. SMAC binds to XIAP, thereby blocking the caspase-inhibiting potential of XIAP. MOMP is also initiated by the extrinsic apoptotic pathway through BID cleavage by caspase-8. Furthermore, cells can die via pyroptosis through DAMP recognition by TLRs leading to inflammasome activation and subsequently caspase-1 activation. Caspase-1 cleaves gasdermins (GSDM) of which the N-terminal cleavage fragments form pores in the plasma membrane. In addition, caspase-1 cleaves pro-IL-1β and pro-IL-18 into their mature forms that are released via GSDM pores.

Mitochondrial apoptosis is regulated by pro- and anti-apoptotic BCL-2 family members. Upon pro-apoptotic stress, activated BAX and BAK induce mitochondrial outer membrane permeabilization (MOMP). MOMP causes the release of soluble intermembrane space proteins, including cytochrome *c*, SMAC and OMI, into the cytosol. Cytochrome *c* binds apoptosis protease activating factor 1 (APAF1) leading APAF1 to oligomerise into a heptameric structure called the apoptosome that activates initiator caspase-9. Active caspase-9 cleaves and activates the effector caspases caspase-3 and caspase-7 leading to rapid cellular demolition (Fig. 2) [26].

Apoptosis can also be engaged through activation of death receptors (extrinsic pathway), such as the TNF and TRAIL receptor, at the plasma membrane. Complex formation at the death receptors allows for the activation and cleavage of caspase-8 which initiates cell death through cleavage and activation of caspase-3 and -7 or MOMP through the cleavage of pro-apoptotic BID into tBID [27–29]. Interestingly, tBID has recently been found to induce MOMP independently of BAX and BAK [30].

Everyday, billions of cells die in our bodies, requiring effective, non-immunogenic clearance to maintain tissue homoeostasis. Apoptosis allows the cell contents to be encapsulated in apoptotic bodies which will be engulfed by phagocytes. Phagocytosis of dying cells, termed efferocytosis, prevents the release of cellular DAMPs, such as lactate dehydrogenase, HMGB1, and ATP, thereby maintaining an immunosilent environment even in the presence of extensive cell death. Failure to clear apoptotic bodies by efferocytosis leads to secondary necrosis and the release of immunogenic DAMPs [31, 32]. In addition, apoptotic cells create an anti-inflammatory microenvironment through the release of immunosuppressors such as interleukin-10 (IL-10), TGF-β, and PGE_2_ [33–35].

## Apoptotic caspases are key determinants of the inflammatory output of cell death

Caspase activity is essential for the execution of apoptotic hallmarks, nonetheless under caspase-inhibiting conditions cells still die in response to widespread MOMP [26]. MOMP causes extensive mitochondrial dysfunction by progressively diminishing mitochondrial respiration [36], causing MOMP to be the point of no return in intrinsic apoptosis.

It is estimated that over 1500 caspase substrates exist in the proteome [37], including regulators of immune signalling pathways and DAMP expression. Inhibition of caspases after MOMP increases NF-κB and type I interferon responses leading to increased pro-inflammatory cytokine production [38–40]. Active caspases cleave and silence DAMPs and key regulators of immune pathways. For example, the DAMP IL-33 is cleaved by caspase-3 and -7 leading to its inactivation [41]. In addition, caspase-dependent cleavage of cGAS, mitochondrial antiviral signalling protein (MAVS), interferon regulatory factor 3 (IRF3), NF-κB essential modulator (NEMO), and IκB kinase β (IKKβ) blocks type I interferon responses and NF-κB signalling [42–44]. Furthermore, caspases also directly interfere with the production of immunogenic proteins through caspase-dependent cleavage of the initiation factors eIF4G, eIF2B and eIF2α, thereby blocking cap-dependent protein translation [45].

Caspases not only play a role in the regulation of MOMP-induced inflammation, but also regulate the immune response during death receptor induced cell death. Delayed cell death and increased cytokine and chemokine production is observed upon combination treatment with caspase inhibitors and death receptor ligands Fas or TRAIL [46, 47]. In addition, the ‘find me’ signals produced during Fas-induced cell death were upregulated during caspase inhibition and promoted phagocyte chemotaxis [46]. Activation of death receptors can lead to MOMP via cleavage of BID into tBID, however these papers do not discuss a role for mitochondrial permeabilization in inflammation through death-receptor mediated apoptosis.

## Release of mtDNA by expanding BAX/BAK pores induces a type I interferon response

**Fig. 3:**
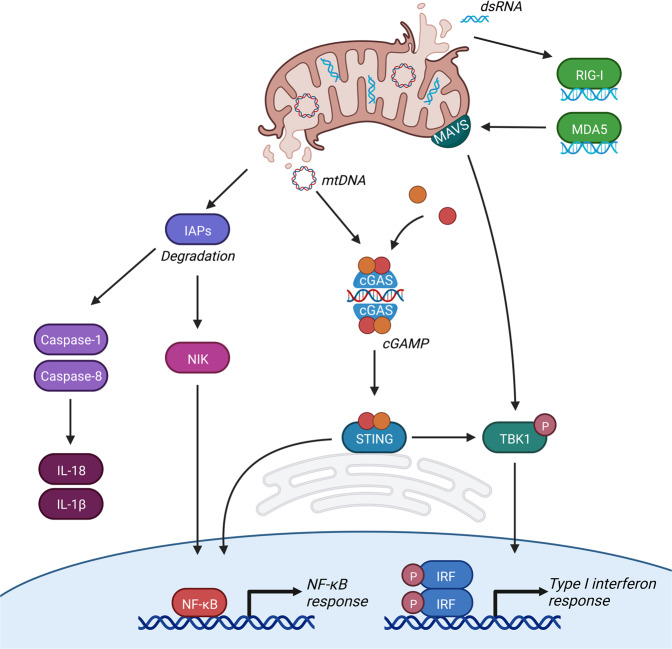
MOMP-induced inflammation. MOMP activates several pro-inflammatory pathways. (1) Under caspase-inhibited conditions MOMP causes IAP degradation which subsequently leads to NIK stabilisation and accumulation followed by the transcription of NF-κB target genes. In addition, degradation of IAPs activates caspase-1 and caspase-8 leading to processing and release of IL-1β and IL-18. (2) Cytosolic release of mtDNA leads to recognition of cGAS which subsequently forms cGAMP out of GTP and ATP. cGAMP is a second messenger for ER-resident STING initiating its activating and the subsequent transcription of NF-κB target genes and type I interferons. (3) Release of cytosolic dsRNA leads to its recognition by RIG-I and MDA5, followed by activation of mitochondria-localised MAVS and a type I interferon response.

Under caspase-inhibited conditions, mitochondrial apoptosis induces a type I interferon response initiated by the detection of cytosolic mtDNA (Fig. 3) [39, 40]. Our lab and others have demonstrated that mtDNA is released through the formation and gradual expansion of BAX/BAK pores during mitochondrial apoptosis [48–51]. However, BAX and BAK are only known to form pores in the outer mitochondrial membrane, leaving the inner membrane intact as observed using electron microscopy [50]. How mtDNA is released from its encapsulation by the inner mitochondrial membrane into the cytosol is currently unknown. Assembly of BAX/BAK pores determines how fast mtDNA will be released. Activation of BAK rapidly causes pore formation and BAX recruitment, yet the incorporation of BAX molecules, which slows down pore formation, eventually results in a larger pore size [49]. Although the formation of BAX/BAK pores results in efficient permeabilization and therefore mitochondrial cell death, the pores formed by either BAX or BAK are sufficient to induce MOMP independently of the other.

The presence of DNA in the cytosol, either nuclear, mitochondrial, or pathogenic, serves as a cellular warning signal caused by pathogen infection or cellular dysfunction. Detection of cytosolic DNA by cGAS enables its dimerisation and subsequent generation of the second messenger cGAMP by using ATP and GTP. Generation of the dinucleotide cGAMP allows activation of the endoplasmic reticulum resident protein STING [13–15, 52–57]. Upon its activation STING translocates to the Golgi where it activates TANK-binding kinase 1 (TBK1) leading to STING phosphorylation [58, 59]. Active TBK1 phosphorylates the transcription factor IRF3, leading to its dimerisation, nuclear translocation, and subsequent transcription of interferon stimulated genes [59–61]. Furthermore, activation of TBK1 can also activate the NF-κB pathway through phosphorylation of the IKK complex [62].

Released mtDNA can also be detected by TLR9 and the NLRP3 inflammasome [22–25, 63, 64]. Endosomal TLR9 recognises hypomethylated CpG-rich motifs present in bacterial and mtDNA. Detection of circulating mtDNA by TLR9 appears to be fundamental in the development of non-alcoholic steatohepatitis (NASH). Mice lacking TLR9 were protected against NASH development when on a choline-deficient amino acid-defined diet [63]. Elevated levels of oxidised mtDNA were detected in hepatocytes and plasma of mice and patients. In addition, treatment of TLR9 antagonist reduced NASH symptoms in mice [64]. The NLRP3 inflammasome also detects oxidised mtDNA [22–25]. The NLRP3 inflammasome acts both downstream and upstream of mtDNA release as it facilitates the formation of the mitochondrial permeability transition pore (mPTP) [22], however it is debatable if formation of the much smaller mPTP is sufficient enough to enable mtDNA release into the cytosol [65]. It is currently unknown if detection of mtDNA by TLR9 or the NLRP3 inflammasome plays a role in MOMP-induced immunogenic cell death.

## IAP depletion activates the NF-κB pathway after MOMP

Inhibition of caspase activity after MOMP not only elicits a type I interferon response but also induces activation of the NF-κΒ pathway independent of STING function (Fig. 3). The activation of this pro-inflammatory pathway is mediated through the degradation of inhibitor of apoptosis proteins (IAPs) leading to stabilisation of the NF-κΒ inducing kinase (NIK), phosphorylation and degradation of IκΒα, and subsequent translocation of the NF-κB family member p65 [38]. Degradation of IAPs and NIK stabilisation has been extensively described by drugs called SMAC mimetics [66, 67], nonetheless degradation of IAPs and subsequent NIK accumulation was still observed when caspase-independent cell death was induced in the absence of intermembrane space proteins SMAC and OMI [38]. MOMP-induced depletion of IAPs has also been observed in macrophages resulting in activation of caspase-8 and the inflammasome thereby inducing caspase-1 dependent maturation of IL-1β and IL-18 [68, 69]. How IAPs are degraded in a SMAC-independent manner during caspase-independent cell death remains unknown.

## MOMP-dependent release of dsRNA induces a type I interferon response

Detection of pathogenic or mitochondrial dsRNA in the cytosol is enabled by retinoic acid-inducible gene I (RIG-I) and melanoma differentiation-associated protein 5 (MDA5) receptors which subsequently mediate the accumulation of MAVS on the mitochondrial outer membrane [70]. Aggregation of MAVS leads to the activation and nuclear translocation of IRF3 to mediate an antiviral immune response [71]. Transcription of mtDNA gives rise to an endogenous source of dsRNA that is normally degraded through a mitochondrial complex termed the degradosome. Patients and mice harbouring mutations in the RNA degradosome complex showed elevated levels of cytosolic mitochondrial-derived dsRNA and type I interferon response [72]. The release of mitochondrial dsRNA was completely blocked upon knockdown of BAX and BAK, indicating that dsRNA can be released through BAX/BAK pores. Furthermore, this response was partially dependent on RIG-I activity and was completely abrogated upon knockdown of MAVS and MDA5 [72]. In addition, a type I interferon response was also observed upon generation of double strand breaks in mtDNA causing BAX/BAK-mediated inner membrane herniation of mitochondria. This response was independent of cGAS-STING signalling and was driven by the dsRNA sensors RIG-I and MAVS [73]. These studies suggest that upon MOMP, release of mitochondrial-derived dsRNA can initiate a type I interferon response through dsRNA sensing by RIG-I and MDA5 and subsequent aggregation and activation of MAVS. It is currently unknown how dsRNA, and mtDNA, pass the inner mitochondrial membrane after MOMP.

## Immune responses during bacterial infection: potential pathways for MOMP-driven inflammation

Even though the first steps of mitochondrial formation occurred 1.5 billion years ago, many similarities can still be observed between mitochondria and bacteria. Therefore, it may be possible that deregulation or destruction of mitochondria allows the exposure of bacterial-like mitochondrial DAMPs thereby initiating immunogenic pathways similar to cytosol-invading bacteria. Recently, two novel pathways for cell autonomous immunity after bacterial infection have been described that may be applicable to mitochondrial-dependent inflammation during cell death.

Ubiquitylation of mitochondrial proteins is extensively described for selective autophagic removal of mitochondria through a process called mitophagy [74], however, in recent years, ubiquitylation of cytosol-invading bacteria has also been implicated to induce an innate immune response [75–77]. Exposure of lipopolysaccharide (LPS) on the bacterial surface allows for its ubiquitylation which will be extended by the formation of pro-inflammatory K63- and M1-linked ubiquitin [77]. Formation of these pro-inflammatory ubiquitin linkages is crucial for the recruitment and binding of NEMO and subsequent NF-κB response [75, 76]. The presence of intramitochondrial K48- and K63-ubiquitin linkages on mitochondria has been observed upon mitochondrial permeabilization and was described to be associated with recruitment of endolysosomes [78].

Recently it has also been described that exposure of glycans on the bacterial surface can initiate the activation of a cell autonomous immune response [79–83]. Exposure of glycans recruit galectins to the bacterial surface which in turn promote the recruitment of IFNγ-inducible GTPases termed guanylate binding proteins (GBPs). These GBPs can be ubiquitylated by the bacterial E3 ligase IpaH9.8 and are subsequently degraded by the proteasome, thereby inhibiting anti-bacterial defence [80, 83]. When not degraded, GBPs form complexes with LPS and galectins to initiate the recruitment of caspase-4 to cytosol-invading bacteria. Sensing of cytosolic LPS activates the inflammasome in a non-canonical manner. Activation of caspase-4 results in gasdermin D (GSDMD) dependent pyroptosis leading to the processing and secretion of pro-inflammatory IL-18 [79, 81]. Whether similar processes occur during the permeabilization of mitochondria is unclear, however two studies have described the presence of galectin-3 at mitochondrial-ER sites and its accumulation on mitochondria after pro-apoptotic stimuli [84, 85] indicating that similar mechanisms might be involved.

## Mitochondria as inflammatory platforms in non-apoptotic forms of cell death

Other forms of inflammatory regulated cell death exist including pyroptosis and necroptosis (Fig. 2). Broadly speaking, the role of mitochondria in regulating inflammation in these forms of cell death is less well defined. Necroptosis is a regulated form of caspase-independent cell death that shares many inflammatory and morphological characteristics with passive, non-regulated necrosis. Necroptosis is best described in context of the TNF receptor complex. Under caspase-8 inhibiting conditions engagement of the TNF receptor complex causes formation of the necrosome by activating receptor interacting protein kinase 1 (RIPK1) and RIPK3. RIPK3 phosphorylates and activates mixed lineage kinase domain-like pseudokinase (MLKL). Cells are killed by the oligomerisation of active MLKL causing permeabilization of the plasma membrane and subsequent release of DAMPs [86]. A role for mitochondria in necroptosis is debatable. Mitophagy-induced depletion of mitochondria did not affect kinetics of necroptosis [87], however mitochondrial ROS can facilitate the initiation of necroptosis by enhancing necrosome formation [88, 89]. So far, no role for mitochondria in necroptosis-induced inflammation has been described.

Pyroptosis is often initiated upon pathogen invasion and relies upon activation of the inflammasome signalling pathway [90]. The canonical pyroptotic pathway is initiated by inflammasome assembly upon stimulation of PRRs leading to the activation of caspase-1 by autoproteolytic cleavage. Activation of inflammasome can also occur in a non-canonical manner through detection of cytosolic LPS, leading to the activation of caspase-4 (human) or caspase-11 (mouse). Activation of caspase-1 causes GSDMD cleavage thereby allowing pore formation of the N-terminal domain into the plasma membrane [91–95]. In addition, active caspase-1 can cleave pro-IL-1β and pro-IL-18 into their mature forms allowing release of pro-inflammatory IL-1β and IL-18 through GSDMD pores prior to cell lysis [92]. Current research implies that pyroptosis affects mitochondrial homoeostasis by reducing the membrane potential, deregulating ion homoeostasis, blocking mitophagy, and inducing MOMP [96–98]. In addition, formation of GSDM pores can induce the release of mtDNA directly and independently of cell lysis [99, 100]. However, a type I interferon response seems to be largely dampened during pyroptosis through cleavage of cGAS by caspase-1 and inhibition of the cGAS-STING pathway through potassium efflux via GSDM pores [98, 101]. It is unclear why an antiviral immune response through type I interferons during pyroptosis would be dampened, while simultaneously initiating an acute immune response through the cleavage and secretion of IL-1β and IL-18.

## Minority MOMP: can sublethal caspase activity block MOMP-induced inflammation?

While apoptosis is a potent tumour suppressor mechanism, engaging apoptosis can have oncogenic effects if not executed properly. Under sublethal apoptotic stress only a few selective mitochondria undergo MOMP, a process termed minority MOMP [102]. Our lab recently described that this selective mitochondrial permeabilization is dependent on mitochondrial fitness. Dysfunctional mitochondria block BAX retrotranslocation thereby accumulating BAX on the mitochondria, making them more prone to MOMP under sublethal stress [103]. Following sublethal apoptotic stress, cells rapidly accumulate DNA damage through caspase-activated DNase and the mitochondrial DNase EndoG [102, 104]. DNA damage acquired by sublethal caspase activity causes genomic instability, cellular transformation, and increased tumorigenesis [102, 104]. In addition, sublethal caspase activation has also been implicated in increased invasiveness in melanoma cells through the activation of the JNK pathway [105].

In theory, activation of MOMP-induced pro-inflammatory signalling pathways should also occur upon the induction of minority MOMP. After widespread MOMP, caspase activation blocks the regulation of various pro-inflammatory signalling pathways. During minority MOMP caspase activity is generally not high enough to induce cell death, raising the question if there is a threshold for caspase activity in blocking inflammatory signalling pathways. Recently, three separate studies observed increased inflammation upon the induction of minority MOMP [73, 106, 107]. Pathogenic infections can induce minority MOMP causing caspase-dependent DNA damage and the secretion of pro-inflammatory cytokines [106, 107]. Furthermore, induction of type I interferons was observed upon minority MOMP caused by the formation of mtDNA double strand breaks. In this setting, herniation of the inner mitochondrial membrane through BAX/BAK pores leads to the release of dsRNA which is subsequently sensed by RIG-I and MAVS [73]. In these studies, inhibition of caspase activity had minimal effect on the production of pro-inflammatory cytokines [73, 106], indicating that sublethal caspase activity does not block minority MOMP-induced inflammation. It is currently unknown if inflammation after minority MOMP has any potential benefits for cancer treatment, however many damaging effects of sublethal caspase activity have been observed leading to increased transformation and tumorigenesis, suggesting that apoptotic caspases have a dark side in cancer.

## Mitochondrial-driven inflammation during cell death: function and potential

**Fig. 4:**
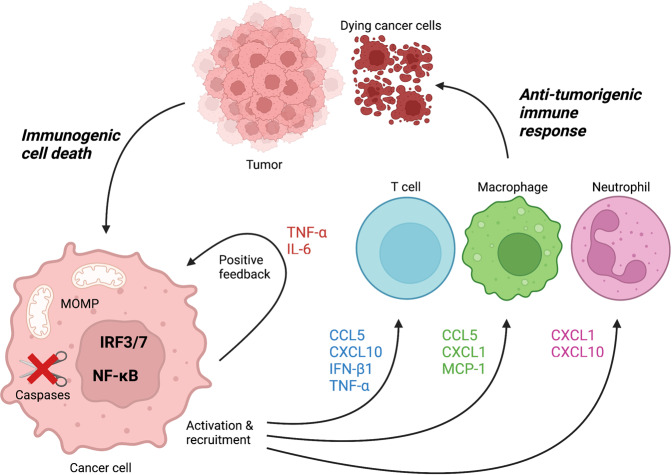
Anti-tumorigenic immune response upon MOMP-induced immunogenic cell death in tumours. Induction of immunogenic cell death in cancer cells can be achieved through MOMP in combination with caspase inhibition. Activation of several cell autonomous immune signalling pathways leads to the transcription and release of type I interferons and NF-κB target genes. Release of cytokines and chemokines recruits and activates anti-tumour T cells, macrophages and neutrophils thereby enhancing cancer cell death and tumour regression.

Emerging evidence in recent years indicates that inducing MOMP-dependent immunogenic cell death, instead of immunosilent apoptosis, has potential benefits in invoking anti-tumour immunity and might therefore be a better strategy for cancer treatment (Fig. 4). For instance, our lab showed that xenografts undergoing MOMP-induced cell death were regressing under caspase-inhibiting conditions but continued to grow out when caspases were active [38]. Here we also established the importance of NF-κB activation in these tumours as xenografts lacking NEMO, an essential component of the IKK complex, did not regress upon the induction of MOMP under caspase-inhibiting conditions. In addition, pharmacological depletion of T cells or the use of immunocompromised mice blocked tumour regression during caspase-independent cell death in xenografts indicating that T cells and an intact immune system are crucial for the anti-tumorigenic properties observed during caspase-independent cell death [38].

Anti-tumorigenic effects of caspase-independent cell death was also observed after irradiation-induced MOMP. Tumour regression of the irradiated primary tumour deficient for caspase-3, as well as regression of distant tumours proficient for caspase-3 was observed [108]. In addition, the importance of a type I interferon response through cGAS-STING signalling was established in xenograft tumours undergoing caspase-independent cell death through irradiation-induced MOMP [109, 110]. Furthermore, the type I interferon response in these tumours upregulated programmed death-ligand 1 (PD-L1) thereby dampening T-cell immunity. Consequently, using PD-L1 inhibitors alongside caspase-independent cell death further enhanced tumour regression observed upon irradiation [109]. In addition, autophagy appears to have a crucial role in dampening the immune response after irradiation as depletion of key autophagy regulators ATG5 or ATG7, thereby blocking mitophagy, increased cytosolic mtDNA after radiotherapy [110]. Irradiation-induced inflammation is dependent on MOMP as BAX deletion abrogated the presence of cytosolic DNA and a type I interferon response. Furthermore, xenograft tumours deficient in ATG5 or ATG7 proved to have a better abscopal response after irradiation which was dependent on a type I interferon response [110].

Unfortunately, applying immunogenic cell death by blocking caspase activity in combination with chemotherapy or irradiation remains a difficult strategy. The pan-caspase inhibitor Emricasan is known to accumulate in the liver and was tested in clinical trials for NASH and liver transplants with limited beneficial effects [111–116]. In addition, long-term treatment with Emricasan had adverse effects by increasing neutrophil infiltration in liver allografts presumably caused by delayed neutrophil apoptosis [114]. Although various studies have shown great potential for immunogenic cell death as a therapeutic strategy for cancer in mice [38, 108–110, 117], no clinical trials have been performed yet to determine treatment efficiency in patients.

## Concluding remarks

In this review we have discussed immunogenic cell death and the role of mitochondria in inflammatory pathway activation. Although mitochondrial apoptosis is considered to be immunosilent, various pro-inflammatory pathways are activated by permeabilised mitochondria but silenced by caspase activity. Initiators of mitochondrial-driven inflammation include the release of mitochondrial dsRNA and mtDNA thereby initiating type I interferons [39, 40, 72, 73]. In addition, depletion of IAPs is associated with a NF-κΒ response after MOMP [38]. Although most eukaryotic cells contain mitochondria there are many variations in the number of mitochondria and formation of the mitochondrial networks, thereby possibly affecting the threshold for mitochondria to undergo MOMP and the quantity of DAMPs that are being released. Traces of the bacterial ancestry of mitochondria are still evident in mtDNA, cardiolipin, and NFPs. Therefore, it may be possible that other initiators of mitochondrial-driven inflammation after MOMP can potentially be found in studies identifying inflammatory pathways upon bacterial infection.

Engaging immunogenic cell death as opposed to immunosilent apoptosis has great potential for cancer therapy. Several studies described that engaging pro-inflammatory types of cell death in tumours improves therapeutic outcomes when compared to the engagement of non-inflammatory cell death [38, 108–110, 117]. Identification of novel pro-inflammatory pathways might help to narrow down key inflammatory pathways to improve cancer therapy by using immunogenic cell death.
